# Genetic Mosaicism as a Cause of Inborn Errors of Immunity

**DOI:** 10.1007/s10875-021-01037-z

**Published:** 2021-04-16

**Authors:** Jahnavi Aluri, Megan A. Cooper

**Affiliations:** Department of Pediatrics, Division of Rheumatology/Immunology, Washington University in St. Louis, 660 S. Euclid Ave. Box 8208, St. Louis, MO 63110 USA

**Keywords:** Inborn errors of immunity, primary immunodeficiency, mosaicism, somatic mutation

## Abstract

Inborn errors of immunity (IEIs) are a heterogeneous group of disorders due to genetic defects in the immune response that have a broad clinical spectrum. Diagnosis of the precise genetic cause of IEI has led to improved care and treatment of patients; however, genetic diagnosis using standard approaches is only successful in ~40% of patients and is particularly challenging in “sporadic” cases without a family history. Standard genetic testing for IEI evaluates for germline changes in genes encoding proteins important for the immune response. It is now clear that IEI can also arise from de novo mutations leading to genetic variants present in germ cells and/or somatic cells. In particular, somatic mosaicism, i.e., post-zygotic genetic changes in DNA sequence, is emerging as a significant contributor to IEI. Testing for somatic mosaicism can be challenging, and both older sequencing techniques such as Sanger sequencing and newer next-generation sequencing may not be sensitive enough to detect variants depending on the platform and analysis tools used. Investigation of multiple tissue samples and specifically targeting sequence technologies to detect low frequency variants is important for detection of variants. This review examines the role and functional consequences of genetic mosaicism in IEI. We emphasize the need to refine the current exome and genome analysis pipeline to efficiently identify mosaic variants and recommend considering somatic mosaicism in disease discovery and in the first-tier of genetic analysis.

## Introduction

Inborn errors of immunity (IEIs) refer to a broad spectrum of genetically heterogeneous group of disorders of the immune system that lead to susceptibility to infection, primary immune regulatory disorders (PIRD), autoinflammation, bone marrow failure, and susceptibility to malignancies [1, 2]. The International Union of Immunological Societies (IUIS) has classified a continuously expanding list of more than 475 primarily monogenic disorders into 9 categories based on the affected branch of the immune system and clinical presentation, with a 10th category based on phenocopies of IEI which includes phenotypes resulting from somatic mutations or due to auto-antibodies [3, 4]. However, evidence for somatic mosaicism (Box 1) as a primary cause of IEIs is also emerging. Understanding the molecular mechanisms of IEI has led to fundamental advances in our understanding of the human immune response, which is critical to the development of treatment methodologies including targeted biologic therapies and hematopoietic stem cell transplantation (HCT), and genetic counseling of patients and families. Current research and clinical methods of genetic diagnosis for patients primarily use exome and genome sequencing, leading to a diagnosis in approximately 40% of patients depending on their age, presentation, and family history [5, 6]. This means that a significant number of patients remain undiagnosed, and new methods for analysis and identification of genetic causes of disease are important for the diagnosis and care of such patients.

Genetic mosaicism has emerged as a significant and often potentially overlooked molecular mechanism of IEI, particularly in patients without a family history of IEI and/or with late-onset presentation who do not have a pathogenic genetic variant identifiable by these standard methods. There are also now several IEIs that were identified solely or primarily as having somatic mosaicism as the cause of disease. In this review, we describe the role of genetic mosaicism in IEI and highlight their increasing contribution to disease pathogenesis of IEI.

Box 1  Genetic terms and definitions

**Table:** 

Genetic term	Definition
Allele	One of 2 or more alternate forms of a gene at the same location. For example, a single variant in *RAG1* in a patient would be considered heterozygous and present on one “allele.”
Coverage	Percentage of targeted genomic regions sequenced to a minimum predefined read depth.
Germline DNA	DNA derived from germ cells (i.e., sperm and egg cells) and present in all cells. Most of our DNA sequence is germline.
Variant	A change in DNA that is different from published reference genome sequence. Variants can be very common, even present in >90% of the population, or rare, for example present in <1% of the population. This is due to the fact that the reference genome was generated from a small number of individuals.
Single nucleotide variant (SNV)	A genetic change in a single nucleotide, for example the change of a guanine (G) to an alanine (A). This may or may not be associated with altered function of the encoded protein, for example by changing the protein sequence or splicing of the mRNA.
De novo variant	A genetic change resulting for the first time in a germ cell or fertilized egg early during embryogenesis. For example, a child with a germline variant not carried by either parent would have a “de novo” variant.
Somatic variant	A post-zygotic change in DNA of somatic cells (i.e., any cell but a germ cell, for example immune cells or skin cells are somatic cells) that is different from the germline DNA. For example, genetic mutations present in leukemia cells are considered “somatic variants.”
Mosaicism	When cells in the same person have different DNA sequences. *Somatic mosaicism* refers to different DNA sequences among somatic cells, for example a genetic change in a subset of immune cells. *Gonadal mosaicism* refers to germ cells having a different sequence than other cells in the same person, for example a mutation in *STAT3* found in sperm but not in other cells. This is different from a de novo variant that can occur in a single germ cell. With *Gonosomal mosaicism*, the genetic variant is present in a portion of both somatic and gonadal cells; in this case, the affected individual may pass the gene on to offspring. This type of mosaicism is due to a mutational event during early embryogenesis.
Reversion mutation	A genetic alteration that reverses the phenotype resulting from the previously mutated gene to wild type functional state. This includes back mutations that restores the wild type sequence or second-site mutations that affect a different site within the protein.
Read depth	Number of sequences computationally aligned to a reference sequence for a given genomic position, for example the number of times a particular fragment of DNA was sequenced. Whole exome sequencing data usually has a “read depth” of at least 100 and whole genome sequencing usually has a “read depth” of at least 30.

## Genetic Mechanisms of IEI and Mosaicism

The majority of IEI disorders are genetically inherited and follow three classic Mendelian modes of inheritance including autosomal recessive (AR), autosomal dominant (AD), and X-linked (XL) [7]. Oftentimes the clinical presentation and family history can help in identifying the possible mode of inheritance in the patient. In AR model of inheritance, two copies of the altered gene are required for disease. In most cases, one disease-causing allele is passed on by each parent, who is typically asymptomatic or may have a subtle phenotype. In such families, there is a 25% chance of having an affected child, for example *RAG1* or *RAG2* deficiency causing severe combined immunodeficiency (SCID). A recessive model of inheritance includes homozygosity or compound heterozygosity of pathogenic variants. Autosomal dominant disorders require one variant allele to cause disease, with a 50% chance of passing the variant and disease on to a child, for example STAT3 dominant-negative variants leading to hyper-IgE syndrome (AD-HIES). XL-IEI are generally due to loss-of-function variants in X-linked genes predominantly affecting males, such as X-linked agammaglobulinemia (XLA). There are exceptions to all of these depending on the molecular defects, for example both AD and AR forms of *IFNGR1* deficiency leading to Mendelian susceptibility to mycobacterial disease (MSMD) [8], or female carriers of *CYBB* variants associated with XL chronic granulomatous disease (CGD) having infectious susceptibility and/or autoimmunity due to non-random X-chromosome inactivation [9]. In addition, digenic causes of IEIs are emerging, with variants in multiple genes leading to new disease phenotypes [6, 10]. For example, a patient was described with severe bacterial and viral infection associated with homozygous deleterious variants in both *IFNGR2* and *IFNAR1*, leading to impaired type I and type II interferon (IFN) responses [11].

**Fig. 1:**
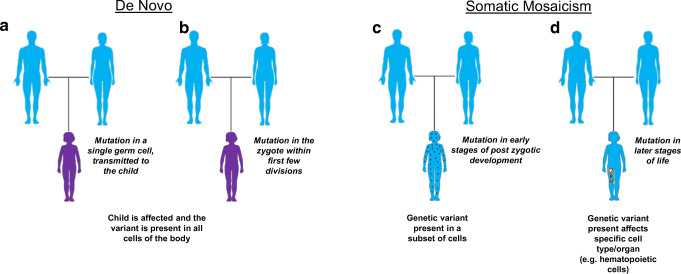
Types of sporadic gene mutations leading to disease. (a, b) De novo variants in affected individuals arising from (a) mutations in paternal or maternal germline cells or (b) post-zygotic mutational events occurring within the first few cell divisions. (c, d) Somatic mosaicism with patients having than one DNA sequence arising from post-zygotic mutations occurring at (c) early stages of embryogenesis, leading to the presence of mutations in a subset of cells from multiple lineages or (d) later stages of development or in adulthood, with mosaicism restriction to a specific cell type/tissue, for example hematopoietic stem cells shown here. Images modified from Servier Medical Art, provided by Les Laboratoires Servier

There are also many cases of seemingly “sporadic” disease, in which an affected child lacks a significant family history and is designated as the first individual in the family to harbor the pathogenic variant. Such variations are referred to as “de novo” variants as they appear for the first time in the family. If the genetic change, or mutation, occurs during the process of meiotic cell division in the female (egg) or male (sperm) germ cells upon conception, it results in an embryo that carries the pathogenic variant in every cell of the body and can be passed on to future generations. In some cases, these variants can also arise from post-zygotic mutations that occur very early in the zygote, within the first few rounds of cell division (Fig. 1a–b). If the variations arise from mitotic errors in the zygote in early stages of post-zygotic development or later stages, the resulting mosaic embryo will carry the variant only in a limited number of cell types or compartment/organ (Fig. 1c–d). Mosaicism refers to the biological phenomenon underlying such genetic alterations that gives rise to the presence of two or more population of cells with different genotypes in an individual [12]. The tissue distribution of the “variant” depends on the stage of embryogenic development at which the mutational event occurs and may affect only the somatic cells (somatic mosaicism), germ cells (gonadal/germline mosaicism), or both (gonosomal mosaicism). This further determines the potential for transmission of the variant to the future generations. For example, a healthy male with a disease-causing variant only in their sperm cells has the potential to pass this variant on to their child, as was described in an interesting case report about a healthy asymptomatic truck driver who fathered multiple children along his route in Brazil, with three half-siblings having activated PI3K delta syndrome 1 (APDS1) due to the same pathogenic variant in *PIK3CD* for which the father had gonadal mosaicism based on sequencing semen [13]. Similarly, an individual with disease caused by mosaicism will not pass the deleterious allele on to their children if it is present only in somatic cells, for example a variant only in hematopoietic cells. In some patients with apparently de novo variants, the variant was inherited from parents who are themselves unknowingly mosaic carriers for the disease-causing allele [14, 15]. Thus, determining the mosaic status in the parents can be important to inform recurrence-risk counseling.

## Identification of Genetic Mosaicism

On the genomic scale, mosaicism can occur as chromosomal alterations including copy number changes, and other structural variants such as large chromosomal deletions, translocations, duplications, and inversions. Most relevant to mechanisms identified thus far in IEI are mosaic variants causing single nucleotide variations (SNVs) or small deletions/duplications [12]. The accurate detection of such mosaicism relies on testing the tissue/cell type that actually harbors the variant and the need for highly sensitive genotyping technologies in cases of low allelic frequency. For example, in patients with cancer, genetic testing can be performed on tumor cells and compared to non-tumor samples to identify somatic variants driving carcinogenesis. However, for IEI it is often not clear what cells drive disease, particularly when trying to identify genetic causes of new diseases.

Multiple tissue types can be used for genetic testing to evaluate for mosaicism, and for IEI, peripheral blood or bone marrow is often the most appropriate since these contain multiple immune cell populations of interest in our patients. It is important to keep in mind that with mosaicism, since not all cells in the sample have the disease-associated genetic change, the frequency of the variant (variant allele frequency or VAF) may be quite low, even less than 5%. The VAF in a sample can depend upon the timing and cell type affected by the mutational event, e.g., occurring during embryogenesis or a later event in a hematopoietic stem cell, and whether the altered allele leads to a survival advantage or disadvantage for the cell. Detection of mosaic single nucleotide variants using traditional methods of molecular screening such as Sanger sequencing is challenging, since the variant peak may be misinterpreted as background noise on the chromatogram due to its relatively small peak height. Molecular technologies that allow for sampling of a high number of products, such as next generation sequencing (NGS), and quantitative PCR techniques including droplet digital PCR can be highly sensitive and efficient techniques for detecting and quantifying mosaicism. Exome and whole genome sequencing are frequently used to identify germline pathogenic variants with coverage of ~100×–200× and 30–60× respectively. While these methods have a much higher limit of detection than Sanger sequencing, they cannot easily detect variants with allelic fractions below 10% [16]. This can be improved by performing increasing overall read depth or targeted sequencing. For example, targeted sequencing of the *NLRP3* gene by amplification of labeled PCR products led to >500x coverage and detected variants with 1% allele fraction in patients with suspected neonatal-onset multisystem inflammatory disease (NOMID) [17]. Another approach is to design a panel to amplify a set of genes relevant to the category of IEI, as recently demonstrated by Mensa- Vilaró and colleagues [14]. In this study, the investigators performed NGS-based method of amplicon-based deep sequencing of a set of ~24 genes, known to cause IEI with a focus on autoinflammatory disorders, at ≥1000× depth. Sequencing was performed on patients from 36 families suspected to have an IEI based on clinical phenotype and negative standard genetic testing. Disease-causing somatic or gonosomal variants were identified in 23 patients (~64%), with a VAF of the apparent disease-causing variant ranging from 0.8 to 40%. One patient was also identified as having a reversion mutation. Parents from another 92 families in which the affected child had an apparent de novo disease-causing germline variant identified that there was actually gonosomal mosaicism in 7% of unaffected parents, highlighting the potential importance of such investigation in determining risk of recurrence in a family. Overall, this study demonstrates the relevance of mosaicism as a cause of IEIs [14].

High-depth (>200×) exome sequencing of purified populations of immune cells may be an approach to identify novel causes of mosaic IEI, particularly if the mutation leads to a selective survival and/or growth advantage for the cell. For example, in VEXAS syndrome (Table 1), somatic variants in *UBA1* were highly enriched in myeloid cells [37]. Even without enrichment of variants in an immune cell population, advances in analysis of sequencing data, such as methods developed to identify somatic variants in tumors [42], have the potential to increase the sensitivity of distinguishing true mosaic variants from sequencing artifact.

**Table 1:** Inborn errors of immunity (IEIs) caused by somatic mosaicism. Diseases here are those in which mosaic somatic variants lead to disease in patients. Not included are variants in asymptomatic individuals (for example gonadal mosaicism) or revertant mosaicism that in some cases can alleviate a disease phenotype. *Indicates disease was initially reported or predominantly mosaic

Disease phenotype		Gene	Chr	GOF or LOF mechanism	Type of mosaicism demonstrated	VAF in blood and/or cell type	References
Autoimmune lymphoproliferative syndrome (ALPS)		*FAS*	Chr10	LOF	Somatic	1–35% in blood (50% in DNTs)	[18–20]
RAS-associated autoimmune leukoproliferative disease (RALD)		KRAS*	Chr12	GOF	Somatic	NA	[21, 22]
		NRAS*	Chr1	GOF	Somatic	50%	[14, 23]
Autoinflammatory disorders	CAPS	*NLRP3*	Chr1	GOF	Somatic	2–45%	[14, 17, 24–30]
	NLRC4 GOF	*NLRC4*	Chr2	GOF	Somatic	30%	[31]
	TRAPS	*TNFRSF1A*	Chr12	GOF	Gonosomal	18–30%	[32]
	Blau syndrome	*NOD2*	Chr16	GOF	Somatic, gonosomal	7–13%	[33, 34]
	SAVI	*TMEM173*	Chr5	GOF	Somatic	NA	[35, 36]
	VEXAS	UBA1*	ChrX	LOF	Somatic	35–80% in blood 60–95% in myeloid cells	[37]
	JAK1 GOF	JAK1*	Chr1	GOF	Somatic	27%	[38]
Hypereosinophilic syndrome		STAT5B*	Chr17	GOF	Somatic	10–46%	[39]
Chronic Granulomatous disease		*CYBB*	ChrX	LOF	Somatic	NA	[40]
Inflammation, neutropenia bone marrow failure, and lymphoproliferation caused by TLR8 (INFLTR8)		TLR8*	ChrX	GOF	Somatic	8–26%	[41]

Abbreviations: *CAPS*, cryopyrin-associated autoinflammatory syndrome; *CINCA*, chronic infantile neurological, cutaneous, and articular syndrome; *DNT*, double-negative T cells; *GOF*, gain-of-function; *LOF*, loss-of-function; *NA*, not available; *SAVI-STING*, associated vasculopathy with onset in infancy; *TRAPS*, tumor-necrosis-factor-receptor-associated periodic syndrome; *VAF*, variant allele frequency; *VEXAS*, vacuoles, E1 enzyme, X-linked, autoinflammatory, somatic

## Mosaicism as a Mechanism of IEI

While we focus here on disease-causing mosaicism in IEI, the first cases of somatic mosaicism reported in IEI during the 1980s and 1990s were reversion mutations that resulted in milder than expected clinical phenotypes in adenosine deaminase deficient (ADA) and X-linked severe combined immunodeficient (SCID) patients [43–47]. In 2004, the first report of an IEI caused by somatic mosaicism was published by Holzelova et al. in describing somatic autoimmune lymphoproliferative syndrome (ALPS) [18], with multiple other discoveries of disease-causing mosaicism since described (Table 1). Many diseases caused by mosaicism are also germline disorders, with both gain-of-function (GOF) and loss-of-function (LOF) mechanisms. An alternative mechanism of LOF can occur when a single germline pathogenic variant does not cause disease or leads to only mild symptoms, but a somatic mutation or acquired loss-of-heterozygosity occurs on the other allele, as sometimes found in patients with low-penetrance germline *FAS* variants [19].

### Autoimmune Lymphoproliferative Syndrome

ALPS is a disorder of lymphocyte apoptosis caused by defects in *FAS*, *FASL*, and their downstream signaling pathways [18]. Patients with ALPS have autoimmunity, most often autoimmune cytopenias, with lymphoproliferation and accumulation of TCRα/β-positive CD4^−^CD8^−^ T cells (double-negative T cells, DNTs) in their peripheral blood and secondary lymphoid organs [48]. Somatic variants in *FAS* (ALPS-sFAS) were initially reported in six unrelated patients with disease that was phenotypically similar to patients with germline AD *FAS* defects [18]. Pathogenic variants in *FAS* were detected in purified double negative T cells (DNTs) with a VAF of ~50%, with low level mosaicism detected in other hematopoietic cells (<10%) but undetectable in non-hematopoietic cells. Analysis of CD34+ hematopoietic progenitor cells (HPCs) in some patients revealed the presence of the mutation in a small number of HPCs, indicating that *FAS* variants originated in these cells [18]. FAS variants are highly enriched in DNTs due to a selective survival advantage conferred to these cells, and patients demonstrate complete disease phenotype despite an overall low percentage of total cells with the disease-causing variant. Somatic *FAS* variants are now known to constitute approximately 15–20% of ALPS cases [20], generally with lymphoid and in some cases also myeloid restriction [18, 19]. Although ALPS-sFAS patients have a disease that in many cases is indistinguishable from patients with germline variants, these patients tend to have a delayed age of presentation [20]. Interestingly, in some families with germline pathogenic *FAS* variants but incomplete penetrance, somatic events in the second *FAS* allele can modulate the disease phenotype [19].

Somatic variants are the primary mechanism of disease for RAS-associated autoimmune leukoproliferative disease (RALD), an ALPS-like disease with non-malignant lymphoproliferation and autoimmune manifestations. Activating somatic mutations affecting codons 12 or 13 in *KRAS* or *NRAS* gene involving myeloid and lymphoid lineages are known to cause RALD [49].

### Autoinflammatory Disorders

Autoinflammatory disorders represent a group of IEIs characterized by innate immune dysregulation with frequent fever, skin findings, and variable organ-specific disease such as arthritis, lung disease, and/or gastrointestinal manifestations [50]. They frequently have dominant molecular mechanisms, increasing the likelihood of a somatic variant causing disease. Cryopyrin-associated periodic fever syndrome (CAPS) causes a syndrome with a varying spectrum of inflammation, fever, rash, and arthropathy due to gain-of-function dominant variants in the *NLRP3* gene [51, 52]. Neonatal-onset multisystem inflammatory disease (NOMID) is the most severe form of CAPS, presenting at birth with CNS inflammation. Although the majority of these patients harbor germline pathogenic variants in *NLPR3*, an international collaborative study demonstrated that 30–40% of patients with negative germline testing harbor somatic *NLRP3* variants [24]. They used a sub-cloning and sequencing strategy and detected mosaicism ranging from 4 to 36%, with similar allelic frequencies in different tissue types tested including peripheral blood immune cells, buccal mucosa, urinary cells, and nail cells, indicating an early mutational event. The sub-cloning and sequencing strategy had a detection limit of ~5% for mosaicism, suggesting that very low-level mosaicism in *NLRP3* may be overlooked using this approach. Saito et al. used a different approach to detect mosaic *NLRP3* variants by stimulating monocytes with lipopolysaccharide (LPS), which selectively led to death in cells with GOF *NLRP3* variants, and sequencing dying monocytes [53]. This approach detected low-level mosaicism (VAF ~ 4.3–6.5%) in 3 NOMID patients with a severe phenotype [53]. Mosaicism as low as 2% in the peripheral blood of CAPS patients was detected using NGS-based amplicon sequencing of the *NLRP3* gene [25]. A challenge of NGS-based technologies is distinguishing sequencing errors from low-level somatic variants. Izawa et al. addressed this by first constructing error-rate maps of NLRP3 amplicons and performing deep sequencing using templates prepared from two-tailed PCR of *NLRP3* exons and a read depth of 350 for each strand, leading to detection down to a VAF of ~1% [17]. Somatic *NLPR3* variants have also been described in patients with a milder disease phenotype and late-onset of presentation (>50 years), with variants restricted to the myeloid compartment [26, 27]. A comprehensive review of somatic and germline *NLRP3* variants revealed that there are “hot spots” for somatic mutation, with only a few variants found in both the germline and somatic state [54]. The phenotypic spectrum of NLRP3 autoinflammatory disease is related to the germline or mosaic status of the variant, with increased severity with germline variants. The authors suggested that that somatic mosaic variants may be incompatible with life if present in germinal state, and similarly germline variants may be asymptomatic when mosaic.

Heterozygous variants in *NLRC4* lead to an inflammasomopathy initially identified as infantile enterocolitis [55]. Kawasaki et al. described an infant with NOMID phenotype where exome sequencing of his genomic DNA identified a mosaic variant in *NLRC4* [31]. Since NOMID had not been associated with germline *NLRC4* variants, they differentiated patient-specific induced pluripotent stem cell (iPSC) lines into myeloid cells to determine pathogenicity of the variant. The iPSC-derived myeloid cells with the *NLRC4* variant produced high levels of IL-1β that normalized when *NLRC4* was gene-edited [31].

Beck et al. [37] recently identified three different somatic variants in *UBA1*, an X-linked gene that encodes for ubiquitin-activating enzyme 1, as a cause of a late-onset, treatment-refractory autoinflammatory syndrome with hematologic abnormalities in 25 unrelated adult males over the age of 45. Variants in this disorder, termed VEXAS (vacuoles, E1 enzyme, X-linked, autoinflammatory, somatic) syndrome, were restricted to the myeloid compartment. To identify this new disease, they used a genotype-driven approach by analysis of exomes and genomes from >2000 individuals with autoinflammatory disease to identify variants in a common gene. Importantly, they considered non-germline variants, in particular on the X-chromosome where such variation is often thought to be a sequencing artifact. Sequencing of purified cell populations revealed that the mosaic *UBA1* variants were present in more than half of hematopoietic progenitor cells and myeloid lineage cells, but were absent in mature lymphocytes and fibroblasts. Functional studies demonstrated that the variants caused a catalytically impaired isoform of UBA1 with decreased ubiquitination leading to activated innate immune pathways. Knockout of the cytoplasmic UBA1 isoform homologue in zebrafish caused systemic inflammation, demonstrating pathogenicity in a model organism [37]. VEXAS is one of a small but growing number of IEI initially identified due to somatic mosaicism (Table 1).

Gruber et al. [38] recently described a patient with early-onset multi-organ immune dysregulation and autoinflammation associated with a mosaic variant in *JAK1*, the gene encoding the tyrosine kinase JAK1, an essential component multiple cytokine signaling pathways. Exome sequencing identified the variant with an AF ~ 27% in the peripheral blood, and present in both hematopoietic and non-hematopoietic cells in varying proportions, indicating an early mutational event. The variant was found to increase JAK1 activity and transactivate partnering JAKs, leading to their hyper-phosphorylation. Similar to germline disease associated with JAK1 GOF, the patient had recurrent cutaneous and gastrointestinal inflammatory disease with eosinophilia. In this case, identification of this mosaic mechanism of disease enabled treating the patient with a JAK inhibitor, leading to the rapid resolution of clinical disease [38].

Other examples of autoinflammatory disorders with disease-causing mosaicism in genes known to cause germline disease include Blau syndrome (*NOD2*) [33], tumor necrosis factor receptor associated periodic syndrome (TRAPS, *TNFRSF1A*) [32], and STING-associated vasculopathy with onset in infancy (SAVI, *TMEM173*) [35, 36]. In Blau syndrome and TRAPS, patients had delayed age of presentation and NGS analysis of multiple tissues/cells including sperm helped to identify low frequency somatic variants in the causative genes and confirm parental germline mosaicism. One affected male with a gonosomal *NOD2* variant passed this on to two daughters who presented with a more severe disease [34]. Two SAVI patients with somatic mosaicism in *TMEM173* had a very early age of onset of presentation (~2 months of age) with a severe clinical phenotype including systemic inflammation and vasculitis [35, 36]. Further analysis of the tissue distribution of the somatic variant in one patient revealed its presence in multiple cell types including buccal mucosa cells, immune cells, fibroblasts, and keratinocytes, suggesting an early mutational event contributing to the severe phenotype [35].

### Other IEI Caused by Mosaicism

Two unrelated males with an intermediate hyper-IgE syndrome (HIES) phenotype were identified with mosaic variants in *STAT3* [56], the gene causing AD-HIES due to dominant-negative variants in *STAT3*. Both patients had normal numbers of Th17 cells, frequently low in AD-HIES, but presented with chronic mucocutaneous candidiasis, staphylococcus infections, and elevated IgE. Both patients transmitted the *STAT3* variant to their children, who then harbored the disease-causing variants in their germline and had AD-HIES with a more severe clinical outcome [56]. This is an example of gonosomal mosaicism, in which a parent harbors the variant in their somatic cells and germ cells and can transmit disease to their children. In this case, the parents were also affected, but in many instances, parents are asymptomatic. Gonosomal mosaicism should be suspected in families with unexpected recurrence of an AD disease.

Somatic GOF variants in *STAT5B* cause a hypereosinophilic syndrome without a known germline equivalent with this gene [39]. One patient with *STAT5B* GOF had relatively mild symptoms of diarrhea, urticaria, and dermatitis with eosinophilia, while the second patient required hematopoietic cell transplantation (HCT). Both patients harbored the same mosaic variant which was detected in all purified immune subsets, with a VAF that varied from >40% in T cells and eosinophils, to ~10% in B cells and dendritic cells. It is unclear why the patients had such distinct clinical phenotypes, suggesting that there may be an influence of other genes and/or environment. Functional studies demonstrated increased STAT5B phosphorylation in patient T cells, helping to establish altered function of the variant [39].

Chronic granulomatous disease (CGD) is a disorder of neutrophil function caused by defects in genes encoding components of the NADPH-oxidase system. Germline variants in the *CYBB* gene cause X-linked CGD. While generally affecting males, women with unfavorable X-chromosome inactivation can have symptoms including infection and autoimmunity, and Wolach et al. [40] identified an older adult woman with severe infections and a somatic variant in *CYBB* present in DNA from white blood cells but not buccal cells. Her neutrophils showed abnormal NADPH oxidase activity, and interestingly there was evidence of extreme skewing of X-chromosome inactivation with only variant mRNA expressed in her white blood cells [40].

We recently described somatic mutations in the *TLR8* gene as a primary mechanism of monogenic IEI [41]. We identified six unrelated boys with neutropenia, B cell defects, and lymphoproliferation, all with novel genetic variants in *TLR8*, the gene encoding toll-like receptor 8 (TLR8), an endosomal TLR that recognizes single-stranded RNA. The clinical phenotype of these patients overlaps with other IEI, including autoimmune lymphoproliferative syndrome, and other primary immune regulatory disorders [57]. The consistent refractory neutropenia with lymphoproliferation makes patients with TLR8 variant a unique entity among IEI. Interestingly, the variants were mosaic in 5/6 patients, with four patients harboring the same mosaic variant. The sixth patient had a different de novo germline variant, and died at a young age due to severe fungal infections. In patients with mosaicism, a VAF of 8–26% was detected in the peripheral blood with similar allele frequencies in sorted immune cells, saliva, and fibroblast lines (5–30%). The variant was not detected in the fibroblasts of one patient, suggesting mosaicism was restricted to the hematopoietic compartment in that patient. The level of mosaicism did not appear related to disease severity, as evidenced by the four patients with the same *TLR8* variant (p.P432L). All four patients had mosaicism in their blood ranging from 14 to 26% VAF; however, one patient was diagnosed with severe disease at age 1 year and died at age 4 years (18% VAF), while the others patients were diagnosed at ages 5, 15, and 16 years with survival into adulthood. Patients had highly activated T cells and a deficiency of class-switched memory B cells on immune phenotyping, with a hyper-inflammatory cytokine signature in their sera. Functional testing in cell lines and patient-specific iPSC derived cells determined that the *TLR8* variants were gain-of-function with increased activation of NF-κB and cytokine production following ligand binding. Three patients had to undergo HCT due to evolving bone marrow failure. Since mosaicism of <30% was sufficient to cause the disease, we anticipate that full donor chimerism may be important for HCT. This was evidenced by one patient who had recurrence of neutropenia with low donor chimerism, which resolved when full donor engraftment was once again achieved following treatment with donor lymphocytes [41]. *TLR8* has not been previously associated with IEI, and our discovery again suggests that mosaicism should be considered in the “first-line” approach of genetic analysis.

### Somatic Reversion Mutations in IEI

In some cases, somatic mutations can partially or fully compensate for a genetic defect. Somatic “back mutations” can restore the wild-type sequence of a pathogenic allele, or second-site compensatory mutations can reverse the effect of the primary disease-causing variant, with both types of “reversion mutations” observed events in IEI [58, 59]. Reversion mutations are especially frequent in Wiskott Aldrich syndrome (WAS) with an estimated incidence of 10% [60]. These mutations offer a substantial selective advantage to revertant cells over mutated cell populations, supporting the accumulation of revertant WASp-expressing cells over time. T cells are the most common lymphocyte population to harbor revertant cells in WAS, with a reversion rate of 5–80% [61–64]. Other primary T cell defects reported with somatic reversions include adenosine deaminase (ADA) deficiency, IL2RG deficiency, and CD3-ζ (CD247) deficiency [65–67]. In these patients, genetic reversions respectively resulted in restoration of ADA enzyme activity to carrier levels, normal expression of the common γ chain of cytokine receptors (γc) in lymphoid, and recovery of CD247 expression and T-cell antigen receptor (TCR) expression. These patients showed progressive clinical improvement after a history of severe clinical course early in life, although not all patients with reversion mutations have significant recovery. Reversion mutations have also been identified in other IEI, including in patients with leukocyte adhesion deficiency type-1 (LAD-1) [68], X-linked lymphoproliferative disease (XLP) [69, 70], and *DOCK8* deficiency [71, 72].

## Challenges in Functional Validation of Mosaic IEI

The identification of a potentially disease-causing mosaic variant in a patient with a suspected IEI comes with challenges to validate pathogenicity of the genetic change. This can be particularly challenging due to varying disease phenotypes based on VAF and cell types harboring the variant. For cases in which the mosaic variant is in a gene known to cause IEI and matches the patient phenotype, functional validation may not be required. However, when a mosaic variant is identified in a gene not known to cause disease, or as in the case of NLRC4 discussed here, known to cause disease but with a different clinical phenotype, functional testing is critical to establish causality. Potential methods to functionally validate mosaic variants in IEI include some of those used for germline disease, including in vitro expression assays to determine whether there is altered function of the encoded protein, or utilizing model organisms to demonstrate that the altered protein leads to a phenotype consistent with the disease [1].

**Fig. 2:**
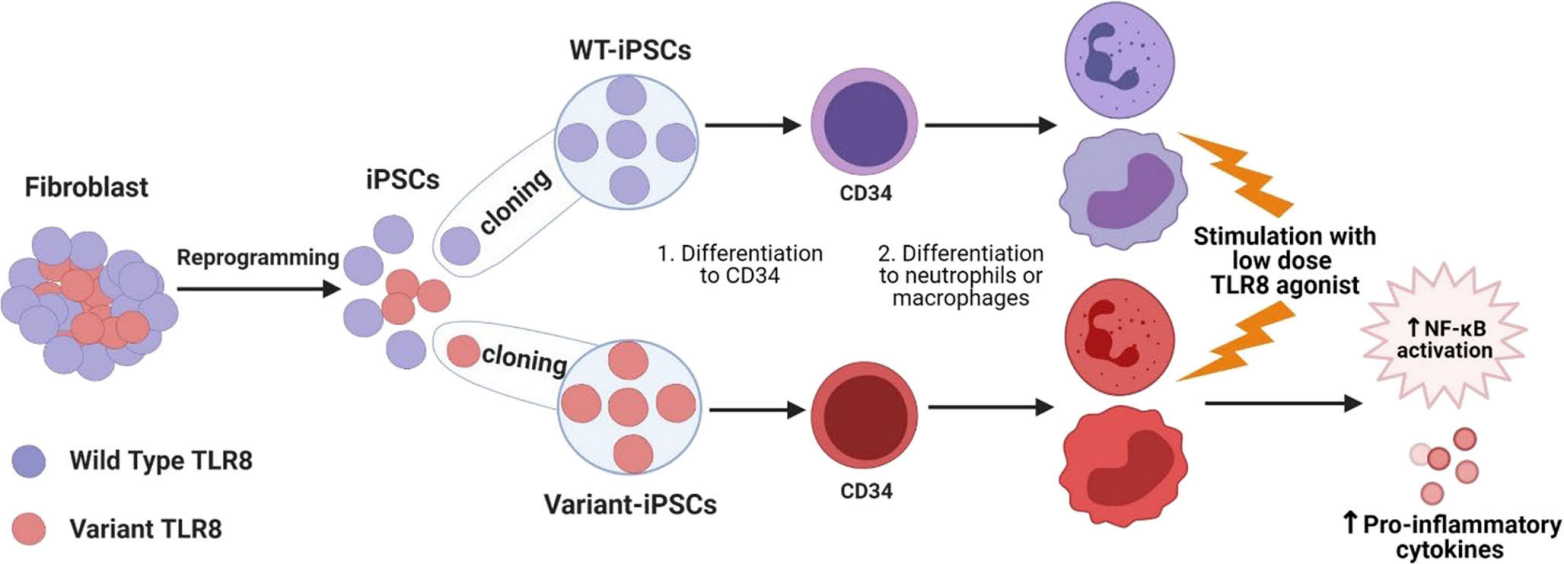
Application of patient-specific induced pluripotent stem cells (iPSCs) for functional analysis of *TLR8* mosaic variants. Cultured skin fibroblasts derived from patients with mosaic *TLR8* variants were reprogrammed into induced pluripotent stem cells (iPSCs), which were single cell cloned to generate clonal iPSC lines with wild type (WT) or TLR8 variant. These clones were differentiated into neutrophils or macrophages and tested for their response to high or low doses of TLR8 stimulation [41]. There was no difference in response to stimulation with a high-dose of TLR8 ligand. Upon stimulation with a low-dose of TLR8 ligand, cells derived from patient-specific iPSC with the *TLR8* variant had increased phosphorylation of NF-κB, and produced high amounts of pro-inflammatory cytokines, as compared to WT iPSC-derived cells, demonstrating a gain-of-function (GOF) phenotype in patient-derived cells. Figure created using Biorender (https://biorender.com/)

Working with primary patient samples can be challenging, as it may be difficult or impossible to phenotypically separate cells carrying the variant in question from cells with a wild-type allele. If the variant leads to loss of protein, it may be possible to distinguish cells based on conventional techniques such as flow cytometry, as shown in a study in patients with somatic ALPS where Janda et al. identified B cells with or without Fas [73]. In some cases, the variant may be enriched in a particular immune cell type, as is the case with somatic ALPS, in which FAS variants are highly enriched for in DNTs. In that case, a cell population can be isolated for functional testing. However, in most cases, the variant is found in multiple lineages and the relevant cell type for the disease is uncertain. For example, in patients with mosaic TLR8 GOF, variants were detected in all immune subsets and it was not possible to separate cells with or without the variant using flow cytometry or other conventional methods. To overcome this, we utilized patient-derived iPSC clones, derived from fibroblasts mosaic for *TLR8* variants (Fig. 2). Clonal iPSC lines with the *TLR8* variant or wild-type (healthy) *TLR8* were generated and differentiated into macrophages and neutrophils, immune cells known to express TLR8 protein. This allowed for direct functional comparison of immune cells with the *TLR8* variant or with wild-type *TLR8* from the same patient in a relevant cell type. This demonstrated increased cytokine production and responsiveness to TLR8 stimulation, confirming the disease-causing gene in these patients. In the case of a patient with a somatic *JAK1* variant, the authors took advantage of the variant expression in B cells to generate clonal EBV lines expressing either WT or *JAK1* variant and establish the variant’s hyper-responsiveness to different cytokine stimuli [38]. They also used custom single-cell RNA sequencing, and sequence data from JAK1-targeted libraries to specifically map and evaluate transcriptomic signatures of cells with the somatic variant. This is a beautiful example of the utility of the growing number of single cell omics, for example single cell RNA-seq, epigenetic profiling, and even proteomics, to evaluate functional responses in individual cells. Combining genotyping with such techniques to assign cells with or without the variant in question has the potential to link genetic and functional responses, and will be important for our understanding of genetic mosaicism in IEI.

## Conclusion

Genetic discovery in IEI has led to more precise diagnosis and treatment of affected patients and an appreciation of the diversity of the human immune response. With increased investigation of patients with rare immune phenotypes, there has been growing recognition that genetic mosaicism can be the underlying causes of IEI. The overall number of patients with IEI caused by post-zygotic somatic mosaicism was previously thought to be relatively low based on isolated case reports in the literature. However, multiple studies, including targeted sequencing for known IEI and discovery of new diseases, have identified somatic mosaicism as an important mechanism of disease. Detection of mosaic variants is challenging in a routine clinical setting due to limitations in available genetic testing approaches, including exome sequencing, and difficulties interpreting functional testing when a disease-causing variant is not present in all cells. As with any genetic disorder, genetic counseling is important for individuals with mosaic disease, as there is the risk of passing the altered allele on to future generations, as in gonosomal mosaicism. Diagnosis of mosaicism can also affect disease treatment, for example, an understanding of donor chimerism required for HCT as we observed with TLR8 GOF. Together, studies of mosaicism in IEI highlight the need for integration of new sequencing approaches into diagnostic algorithms, particularly for patients lacking a germline disease-causing variant in which there is a strong clinical suspicion for an underling IEI. This will eventually lead to a better management of patients with IEI and discovery of new diseases caused by somatic variants.

## Data Availability

Not applicable
